# Agonist anti-GITR antibody significantly enhances the therapeutic efficacy of *Listeria monocytogenes*-based immunotherapy

**DOI:** 10.1186/s40425-017-0266-x

**Published:** 2017-08-15

**Authors:** Rajeev Shrimali, Shamim Ahmad, Zuzana Berrong, Grigori Okoev, Adelaida Matevosyan, Ghazaleh Shoja E. Razavi, Robert Petit, Seema Gupta, Mikayel Mkrtichyan, Samir N. Khleif

**Affiliations:** 1 Augusta University, Georgia Cancer Center, 1410 Laney Walker Blvd, Augusta, GA 30912 USA; 2 Advaxis Immunotherapies, Princeton, NJ 08540 USA

**Keywords:** Listeria vaccine, Lm-LLO-E7, Anti-GITR antibody, Co-stimulation, Immune tolerance, Immunotherapy

## Abstract

**Background:**

We previously demonstrated that in addition to generating an antigen-specific immune response, *Listeria monocytogenes* (Lm)-based immunotherapy significantly reduces the ratio of regulatory T cells (Tregs)/CD4^+^ and myeloid-derived suppressor cells (MDSCs) in the tumor microenvironment. Since Lm-based immunotherapy is able to inhibit the immune suppressive environment, we hypothesized that combining this treatment with agonist antibody to a co-stimulatory receptor that would further boost the effector arm of immunity will result in significant improvement of anti-tumor efficacy of treatment.

**Methods:**

Here we tested the immune and therapeutic efficacy of *Listeria*-based immunotherapy combination with agonist antibody to glucocorticoid-induced tumor necrosis factor receptor-related protein (GITR) in TC-1 mouse tumor model. We evaluated the potency of combination on tumor growth and survival of treated animals and profiled tumor microenvironment for effector and suppressor cell populations.

**Results:**

We demonstrate that combination of *Listeria*-based immunotherapy with agonist antibody to GITR synergizes to improve immune and therapeutic efficacy of treatment in a mouse tumor model. We show that this combinational treatment leads to significant inhibition of tumor-growth, prolongs survival and leads to complete regression of established tumors in 60% of treated animals. We determined that this therapeutic benefit of combinational treatment is due to a significant increase in tumor infiltrating effector CD4^+^ and CD8^+^ T cells along with a decrease of inhibitory cells.

**Conclusion:**

To our knowledge, this is the first study that exploits Lm-based immunotherapy combined with agonist anti-GITR antibody as a potent treatment strategy that simultaneously targets both the effector and suppressor arms of the immune system, leading to significantly improved anti-tumor efficacy. We believe that our findings depicted in this manuscript provide a promising and translatable strategy that can enhance the overall efficacy of cancer immunotherapy.

## Background

Many of the immune inhibitory and costimulatory receptors or their ligands are expressed on both immune suppressive cells such as regulatory T cells (Tregs), as well as effector immune cells and antigen presenting cells (APCs) fostering a complex level of interaction within the tumor microenvironment. Effector T cells and Tregs express various co-stimulatory receptors including 4-1BB, OX40, GITR, CD270, CD27, TNFRSF25 and CD40, which are members of the tumor necrosis factor receptor (TNFR) superfamily. Monoclonal antibodies targeting these receptors have been developed to activate the immune system by acting as an agonist with proven therapeutic potential in different tumor models [1, 2]. Given the dynamic and complex nature of tumors, it has been suggested that targeting either immune inhibitory receptor–ligand interactions, such as CTLA-4 and PD-1 to break immune tolerance, and activating the costimulatory receptor-ligands to enhance the effector immune response, may potentiate the anti-tumor immune response with eventual therapeutic benefits in certain types of tumor models.

Among the costimulatory targets, glucocorticoid-induced tumor necrosis factor receptor-related protein (GITR, TNFRSF18, and CD357) is one of the key members of the TNFR superfamily, that is generally expressed on a wide variety of immune and non-immune cells, including Tregs, effector T cells, natural killer cells, B cells, macrophages, dendritic cells, keratinocytes, neurons, bone marrow stromal cells and mircoglia [2, 3]. Upon engagement with its ligand, GITR and other members of the TNFR family promote activation, expansion and survival of T cells, enhance effector function activity including cytokine production, generate T cell memory and potentiate the inflammatory response in favor of tumor rejection. GITR is expressed at a low level on resting CD4^+^ and CD8^+^ T cells and is constitutively expressed on Tregs [2, 4, 5]. Compared to resting T cells, it has been observed that Tregs strongly and preferentially proliferate upon GITR binding, which further suggests a strong costimulatory effect of GITR on Tregs compared with resting CD4^+^ and CD8^+^ T cells [4]. GITR agonist monoclonal antibody (mAb) DTA-1 and sGITR-Ligand, which augments in vitro proliferation of both Tregs and naïve T cells, has been shown to abrogate Treg-mediated suppression of CD4^+^CD25^−^ T cells in the in vitro suppression assay [6, 7]. In support of this, in vivo studies have shown that DTA-1 not only activates effector T cells and mediates depletion of tumor-infiltrated Tregs, but also results in the loss of FoxP3 expression [7, 8]. Taken together, these effects lead to subsequent lineage instability of intra-tumor Tregs and loss of their immune-suppressive function, making the tumor vulnerable to effector T cell-mediated cytotoxicity. Moreover, therapeutic outcome due to direct stimulation of tumor-specific effector CD4^+^ and CD8^+^ T cells by GITR agonist DTA-1 has been demonstrated in various vaccine - tumor models and adoptive transfer studies without affecting the Treg population and their function [3, 9–13]. Notably, it has been demonstrated that GITR signaling does not induce tumor regression unless Tregs are depleted [14]. Accordingly, we hypothesized that combination of anti-GITR antibody (Ab) that enhances the number of effector CD4^+^ and CD8 T^+^ cells with immunotherapies that inhibit suppressive cells such as Tregs or myeloid-derived suppressor cells (MDSCs) will synergize to lead to a potent anti-tumor effect. We previously showed that *Listeria*-based vaccine immunotherapy significantly reduces Treg/CD4^+^ cell ratio in the tumor microenvironment [15]. Indeed, *Listeria*-based vaccines have been successfully exploited in various preclinical and clinical studies due to their unique and dual property inhibiting suppressive cells while simultaneously enhancing the immune response by increasing effector T cell function and antigen-presenting ability of dendritic cells [15–18]. Furthermore, MDSCs, another type of major immune suppressive cells within the tumor microenvironment that can limit the efficacy of immunotherapy, are also known to be regulated by *Listeria*-based vaccine [19, 20]. Therefore, we exploited the Treg-diluting potential of Lm-based vaccine to enhance the therapeutic efficacy of agonist anti-GITR Ab by combining these two treatments in TC-1 tumor-bearing mice.

For the first time, our findings show that agonist anti-GITR Ab and *Listeria*-based immunotherapy exhibits synergism that leads to complete tumor regression in 60% of treated mice and significant inhibition of tumor growth in the remaining animals. We demonstrate that this therapeutic efficacy is dictated by multiple factors including enhancement of both tumor-infiltrating and peripheral antigen-specific effector CD8^+^ T cells, diluting the Treg population within the CD4^+^ T cell pool through enhancement of non-Treg CD4^+^ T cells, and stabilizing the level of suppressive MDSCs within the tumor microenvironment.

We believe that our proposed combination gives an opportunity to simultaneously break the immune tolerance mediated by immune suppressive Tregs and MDSCs while activating the co-stimulatory receptor-ligands to enhance the antigen-specific effector immune response.

## Methods

### Mice

C57BL/6 6–8-week-old female mice were purchased from The Jackson Laboratories and housed under pathogen-free conditions. All procedures were carried out under the guidelines of the National Institutes of Health and in accordance with approved Augusta University IACUC animal protocols.

### Tumor cell line

TC-1 (primary C57BL/6 mouse lung epithelial cells) that were derived by stable co-transfection of human papillomavirus strain 16 (HPV16) early proteins 6 and 7 (E6 and E7) and activated h-ras oncogene were obtained from Dr. T-C Wu (Johns Hopkins University) [21]. Cells were grown in RPMI 1640 supplemented with 10% FBS, 2 mM L-glutamine, penicillin (100 U/ml) and streptomycin (100 μg/ml) at 37 °C with 5% CO_2_ and maintained at a confluence of 70%–80%.

### Listeria-based vaccine

Highly attenuated *Listeria*-based vaccine vectors with or without LLO and HPV-16 E7 (Lm, Lm-LLO and Lm-LLO-E7) provided by Advaxis Inc. were generated similarly to the previously described method [15, 17]. Lm, Lm-LLO and Lm-LLO-E7 were injected intraperitoneally (i.p.) at 5 × 10^6^ CFU/mouse dose.

### Antibodies and reagents

Anti-mouse GITR Ab (DTA-1 clone) was obtained from Biolegend (San Deigo, CA). Live/Dead Fixable red dead cell stain kit was obtained from Molecular Probes, NY. Flow cytometry antibodies to CD45, CD3, CD4, CD8, CD11b and GR1 were obtained from BD Biosciences. Intracellular FoxP3 staining kit was from eBiosciences and E7- FITC dextramers were from Immudex.

### Tumor implantation, immunization, Ab treatment and tumor volume measurement

Mice were implanted with 70,000 of TC-1 cells/mouse subcutaneously (s.c.) into the right flank on day 0. Ten to 12 days (D10-D12) later when tumors measured ~5-6 mm in diameter, mice from appropriate groups (10 mice per group) were injected i.p. with Lm, Lm-LLO, or Lm-LLO-E7 with or without anti-GITR Ab. Mice were given a total of two doses of vaccine within a seven-day interval. Anti-GITR Ab was given at a dose of 5 mg/kg (100 μg/dose) at 3–4 day intervals for a total of four doses. Tumors were measured every 3–4 days using a digital caliper, and the tumor volume was calculated using the following formula: V = LxW^2^/2, where V is tumor volume, L is the length of tumor (longer diameter) and W is the width of the tumor (shorter diameter). In these studies, mice were sacrificed when tumors reached 1.5cm^3^ in volume or tumors became ulcerated or when mice became moribund.

### Flow cytometry analysis of spleen and tumor infiltrating lymphocytes and MDSCs

For cellular immune response evaluation, mice were treated following the same schedule as in the therapy experiment. Six days after the second vaccination, mice were euthanized to harvest the spleen and tumor, which were further processed using a GentleMACS dissociator and the solid tumor homogenization protocol, as suggested by the manufacturer (Miltenyi Biotec, Auburn, CA). One million cells were stained with the live and dead staining kit (Molecular Probes) followed by surface Ab staining, fixation and permeabilization according to the manufacturer’s protocol. Intracellular staining kits were used to stain for FoxP3 using anti–FoxP3-APC mAb (eBioscience). Further cells were incubated for 20 min on ice in PBS, 2% BSA, and 0.1% sodium azide with a cocktail of antibodies against the above markers. Data acquisition was performed on a LSRII flow cytometer (BD Biosciences). Results were analyzed with FlowJo software (TreeStar). Total number of CD3^+^, CD8^+^, CD4^+^, CD8^+^E7^+^, FoxP3^+^ and GR1^+^CD11b^+^ were analyzed within the CD45^+^ hematopoietic cell population and represented in 1 × 10^6^ live cells in tumor while in spleen the above subpopulations were directly represented as % of live splenocytes.

### ELISPOT assay

Standard ELISPOT assay was used to detect interferon gamma (IFN-γ) production in E7-restimulated (10 μg/ml) splenocyte cultures from treated and control mice, as suggested by the manufacturer (BD Biosciences, San Jose, CA). Splenocytes harvested during the immune response study were incubated with ACK lysis buffer for 5 min to lyse red blood cells (RBCs). Following RBC lysis, cells were washed, filtered, and counted, and 0.4 × 10^6^ cells were plated along with E7 peptide in anti-IFN-γ-coated plates at 37 °C with 5% CO_2_ for 24 h. Plates were washed, blocked and further developed with anti-IFN-γ detection Ab using biotin-streptavidin-HRP complex followed by AEC substrate (BD Biosciences, San Jose, CA). Finally, plates were washed and allowed to dry for spots to develop. Spots were counted and analyzed using CTL Immunospot Analyzer (Cellular Technology, Shaker Heights, OH), and the results were examined for differences in number of spots between E7 re-stimulated and irrelevant peptide-stimulated splenocyte cultures.

### Statistical analysis

All statistical parameters (average values, SD, SEM, statistical significant differences between groups) were calculated using GraphPad Prism Software. Statistical significance between groups was determined by one-way ANOVA with Tukey’s multiple comparison post-test (*P* ≤ 0.05 was considered statistically significant). Survival in various groups was compared using GraphPad Prism using Log-rank (Mantel-Cox) test. SK plots were generated by internally developed software (https://skylineplotter.shinyapps.io/SkyLinePlotter/). Contrary to the survival plot made using GraphPad Prism, the SK plot gives dynamic simultaneous presentation of tumor volumes and mouse survival at a specific time point.

## Results

### GITR co-stimulation enhances therapeutic potency of Lm-LLO-E7 immunotherapy in TC-1 tumor model

**Fig. 1:**
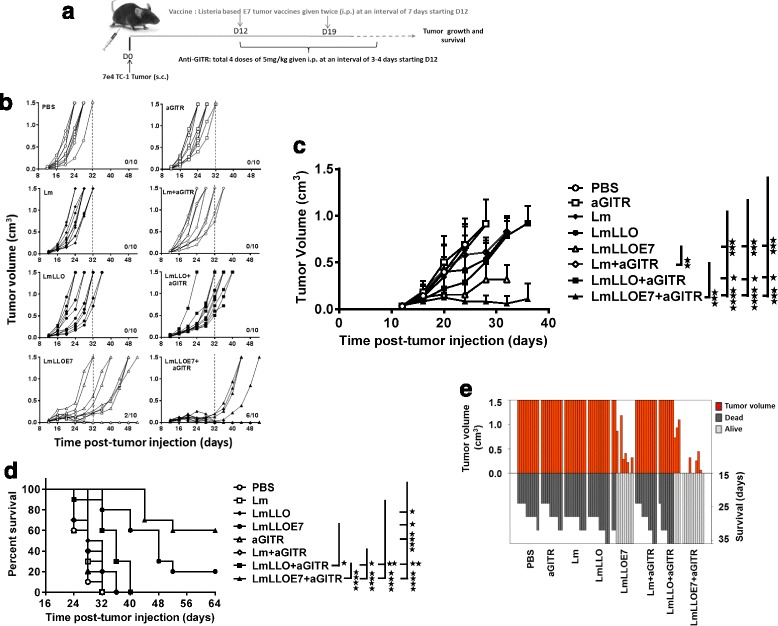
**a.** Schematic of experiment. Starting on day 12 of tumor growth, TC-1 tumor-bearing, 6–8-week-old C57BL/6 female mice (*n* = 10 per group) were given anti-GITR Ab (i.p., 5 mg/kg daily, total 4 doses) along with *Listeria*-based E7 vaccine (i.p., two doses) every 7 days. Tumor growth and survival were measured until the end of the experiment. **b.** Tumor growth for individual mice in each group is presented. The number of mice with completely regressed tumors out of the 10 mice in the group is indicated. **c.** Averaged tumor volumes following various treatments. Statistical significance is shown for day 24. **d.** Kaplan-Meier plot for survival. **e.** SK Plot showing the tumor volume and survival for each mouse at different days. (*P ≤ 0.05, **P ≤ 0.01, ***P ≤ 0.001, ****P ≤ 0.0001)

We evaluated the anti-tumor efficacy of *Listeria*-based immunotherapy in combination with agonist anti-GITR Ab in the TC-1 mouse tumor model. In this therapeutic study, Lm, Lm-LLO or Lm-LLO-E7 was given in two doses within a seven-day interval and anti-GITR Ab was injected intraperitoneally for a total of four doses at 3–4 day intervals. The treatment schema is summarized in Fig. 1a. We found that Lm-LLO-E7 treatment alone exhibited significant anti-tumor activity resulting in inhibition of tumor growth (***P ≤ 0.001) and complete regression of 20% of tumors (****P ≤ 0.0001) in treated mice compared to untreated controls (Fig. 1b-e). While anti-GITR Ab (aGITR) alone did not show any therapeutic benefit, its addition to Lm-LLO-E7 led to further significant enhancement of treatment efficacy, resulting in even more profound inhibition of tumor growth (****P ≤ 0.0001) (Fig. 1b-c) and complete regression of tumors in 60% of animals (****P ≤ 0.0001) (Fig. 1d-e). Furthermore, we re-challenged the mice with completely regressed tumors (and naïve mice as a control) either with double the amount of TC-1 cells or with the irrelevant B16 tumor cells. None of the “cured” mice receiving the TC-1 cells developed tumor, while naïve mice implanted with TC-1 and “cured” mice implanted with B16 cells had tumor growing in normal pace (data not shown). These data demonstrate synergistic activity of *Listeria*-based immunotherapy when combined with agonist anti-GITR Ab in potent inhibition and eradication of tumors.

### Anti-GITR Ab preferentially enhances Lm-LLO-E7-induced CD4^+^FoxP3^−^ cells thereby diluting Treg content in total CD4^+^ T cell pool

We and others have previously reported that Lm-LLO-E7 enhances overall the CD4^+^ effector T cells by preferentially expanding the CD4^+^FoxP3^−^ conventional T cells [17, 22]. Here we made a similar observation in our current immune response study, where mice were treated as in therapeutic studies, but were euthanized 6 days after the second *Listeria*-based treatment to harvest spleens and tumors for immune cell profiling.

**Fig. 2:**
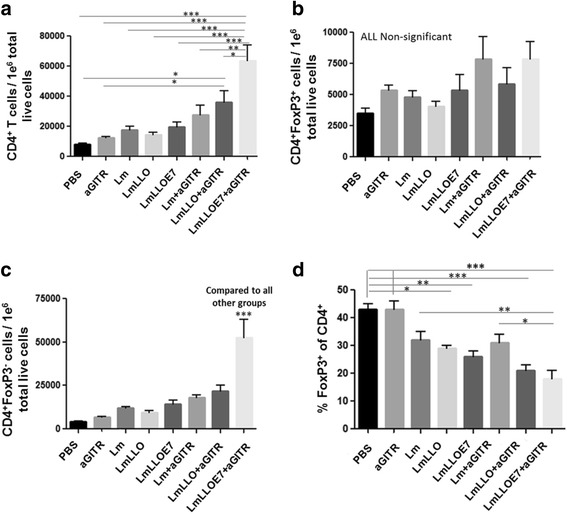
Six days after the second *Listeria*-based immunotherapy treatment, mice were euthanized and tumors harvested and profiled for **a. ** total number of CD4^+^ T cells per 1e^6^ tumor cells; **b.** total number of Tregs (CD4^+^FoxP3^+^) per 1e^6^ tumor cells; **c** total number of non-Tregs (CD4^+^FoxP3^−^) per 1e^6^ tumor cells; and **d.** Percentage of Tregs within the CD4^+^ T cell population. (*P ≤ 0.05, ** *P* ≤ 0.01, ***P ≤ 0.001). Experiment was repeated twice with *n* = 5/group with similar results

We showed that Lm-LLO-E7 slightly, but not significantly, increased the total number of tumor-infiltrating CD4^+^ T cells (Fig. 2a). Adding agonist GITR Ab significantly enhanced overall the CD4^+^ population in the Lm-LLO-E7-treated group (***P ≤ 0.001 Fig. 2a). We did not observe any significant effect on tumor-infiltrating CD4^+^FoxP3^+^ Tregs by any of the treatments compared to the PBS control group (Fig. 2b). Next we showed that adding agonist anti-GITR Ab to Lm-LLO-E7 significantly increased the number of CD4^+^FoxP3^−^ (non-Treg) CD4^+^ T cells compared to all other groups (Fig. 2c). This preferential expansion of CD4^+^FoxP3^−^ T cells with no effect on CD4^+^FoxP3^+^ Tregs resulted in a significant decrease in the proportion/percentage of suppressive Tregs among the total CD4^+^ T cells (Fig. 2d). These data demonstrate synergistic activity of Lm-LLO-E7 and anti-GITR Ab combination in increasing the FoxP3-negative population of CD4^+^ T cells within the tumor microenvironment, resulting in a decrease in the Treg population within the total CD4^+^ T cell pool.

### Anti-GITR Ab enhances CD8^+^ T cell tumor infiltration in Listeria-based immunotherapy recipients while enhancing tumor-infiltrating antigen-specific CD8^+^E7^+^ T cells only in Lm-LLO-E7-treated mice

**Fig. 3:**
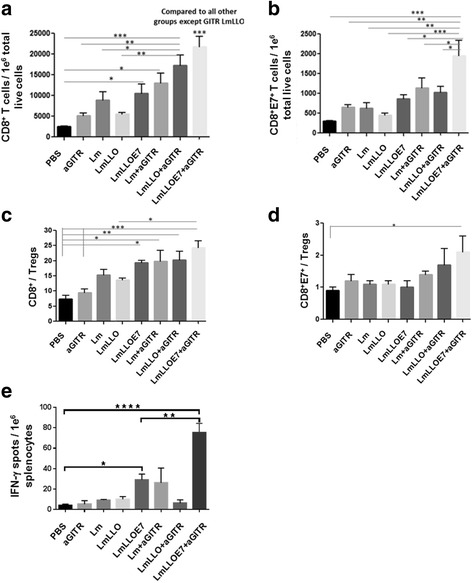
Six days after the second *Listeria*-based immunotherapy treatment, mice were euthanized and tumors harvested and profiled for **a.** total number of CD8^+^ T cells; and **b.** total number of E7-specific CD8^+^ T cells (CD8^+^E7^+^) per 1e^6^ tumor cells. Ratios for CD8^+^/Tregs (**c**) and CD8^+^E7^+^/Tregs (**d**) were calculated. **e.** Six days after the second treatment with *Listeria*-based immunotherapy treatment, mice were euthanized and spleens were collected for ELISPOT analysis. Antigen-specific CD8^+^ T cells in the presence of E7_49–57_ peptide versus irrelevant peptide control was assayed by ELISPOT. Values are presented as number of spots from E7_49–57_ restimulated culture minus irrelevant Ag re-stimulated culture per million splenocytes. (*P ≤ 0.05, **P ≤ 0.01, ***P ≤ 0.001, ****P ≤ 0.0001). Experiment was repeated twice with *n* = 5/group with similar results

Studies from our group have previously reported that *Listeria*-based immunotherapy enhances overall the CD8^+^ effector T cells [17]. In the current study, we observed that Lm-LLO-E7 increases the total CD8^+^ effector T cell infiltration into the tumor, which is further significantly amplified by addition of agonist anti-GITR Ab (***P ≤ 0.001) (Fig. 3a). Interestingly, E7 antigen-specific CD8^+^ (CD8^+^E7^+^) effector T cells were significantly enhanced only in mice treated with a combination of Lm-LLO-E7 and anti-GITR Ab (Fig. 3b). While the CD8^+^ T cell to Treg (CD8^+^/Treg) ratio as a therapeutic index was increased in four treatment groups (LmLLOE7, Lm + aGITR, LmLLO + aGITR and LmLLOE7 + aGITR) compared to the PBS and anti-GITR Ab alone groups (Fig. 3c), the CD8^+^E7^+^/Treg therapeutic ratio was significantly higher (*P ≤ 0.05) only in the group receiving Lm-LLO-E7 vaccine with agonist GITR Ab compared to control (Fig. 3d). These data demonstrating synergistic activity of the Lm-LLO-E7 and anti-GITR Ab combination in increasing the number of antigen-specific CD8^+^ T cells correlated with the results of the therapeutic efficacy studies shown above (Fig. 1b-e).

### Anti-GITR Ab enhances the antigen-specific immune response of Lm-LLO-E7 not only in the tumor microenvironment but also in the periphery

After demonstrating the increase of antigen-specific CD8^+^ T cells within the tumor microenvironment by combinational treatment with Lm-LLO-E7 and anti-GITR Ab, we next asked, if this enhancement has a systemic nature or is restricted to the tumor environment. Thus, we evaluated the number of CD8^+^ T cells within the spleens of treated animals that secrete IFN-γ in response to re-stimulation with CTL epitope from E7 antigen (RAHYNIVTF) using a standard ELISPOT assay.

As expected, we observed a significant increase in antigen-specific CD8^+^ T cells when mice were treated with Lm-LLO-E7 compared to PBS control (Fig. 3e). Importantly, the group receiving the combination of Lm-LLO-E7 and anti-GITR Ab exhibited significantly higher levels of IFN-γ-secreting CD8^+^ cells in response to E7 re-stimulation compared not only to control groups but also to the Lm-LLO-E7 group (**P ≤ 0.01). Results from this study correlate with enhanced antigen-specific CD8^+^E7^+^ T cells within the tumor microenvironment, as well as with data from therapeutic efficacy studies.

### Listeria-based immunotherapy reverses anti-GITR Ab-mediated increase in tumor-infiltrating MDSCs

**Fig. 4:**
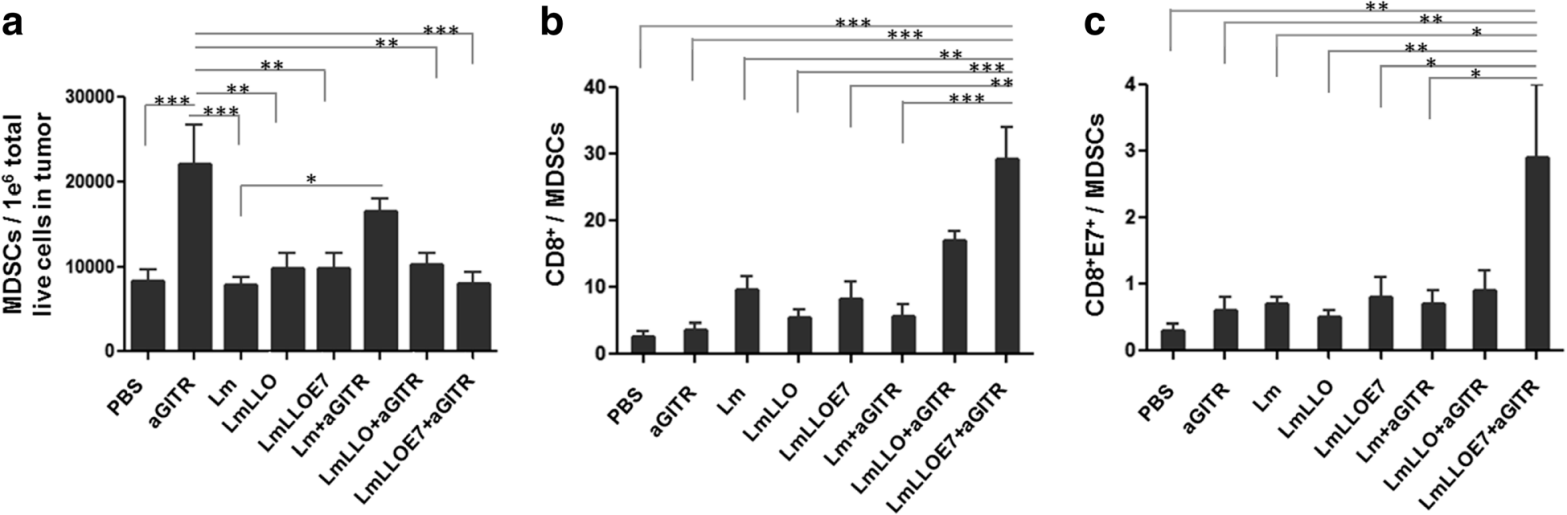
Six days after the second treatment with *Listeria*-based immunotherapy treatment, mice were euthanized and tumors harvested and profiled for **a.** total number of GR1^+^CD11b^+^ MDSCs per 1e^6^ tumor cells. Ratios for CD8^+^/MDSCs (**b**) and CD8^+^E7^+^/MDSCs (**c**) were calculated. (*P ≤ 0.05, ** *P* ≤ 0.01, ***P ≤ 0.001). Experiment was repeated twice with *n* = 5/group with similar results

We and others previously demonstrated that *Listeria*-based immunotherapy can control the number and immune suppressive activity of MDSCs [15, 19]. Here we also evaluated the effect of co-stimulating GITR in the presence of E7-based *Listeria* immunotherapy on the status of tumor-infiltrating MDSCs. We found that treatment with agonist anti-GITR Ab in this model significantly (***P ≤ 0.001) increased the number of GR1^+^CD11b^+^ MDSCs within the tumor microenvironment (Fig. 4a). While we did not observe any significant effect of *Listeria*-based treatment alone on the number of MDSCs, the addition of any type of *Listeria* treatment resulted in a reversal of the anti-GITR Ab-induced increase in MDSCs (Fig. 4a). This inhibition of MDSCs (mediated by *Listeria*) along with enhancement of CD8^+^ T cells (mediated by anti-GITR Ab) resulted in a dramatic increase in both CD8^+^/MDSCs (Fig. 4b) and CD8^+^E7^+^/MDSCs (Fig. 4c) ratios, further explaining the mechanism of synergy of this combination and the beneficial effect of anti-GITR addition to Lm-LLO-E7 seen in the therapeutic studies.

## Discussion

Tregs are one of the key immune suppressive T cells dedicated to control autoimmunity and mediate immunological self-tolerance by exerting a suppressive effect on the effector immune response generated against foreign, mutated or self-antigens, including tumor antigens [23]. For more than a decade, preclinical research and clinical findings demonstrated that Tregs play a major immune suppressive role and their presence has been associated with poor prognosis and tumor recurrence in a wide range of tumor types [24]. Moreover, Tregs have been shown to abrogate effector CD8^+^ T cell-mediated tumor rejection by suppressing the cytolytic activity of the proliferated CD8^+^ T cells through TGF-β-mediated signaling [25].

Tumor vaccines, along with enhancing antigen-specific effector immune cells, have been shown either to induce or to functionally enhance immune suppressive tumor-infiltrating Tregs and MDSCs that can negatively affect effector cell function and overall outcome of tumor vaccine therapy [22]. *Listeria*-based tumor vaccines against various tumor-associated antigens have been presented as a successful tumor immunotherapy approach in both preclinical and clinical settings [26–30]. The *Lm* is a Gram-positive facultative intracellular pathogen that preferentially infects APCs, effectively induces both MHC class I and class II responses, and generates potent cytotoxic T lymphocyte (CTL)-mediated tumor killing. To date, two recombinant Lm-based HPV-16 associated cervical cancer vaccines have been introduced, one that expresses and secretes E7 protein (Lm-E7), and the other, Lm-LLO-E7, secretes E7 as a fusion protein joined to a non-hemolytic listeriolysin O (LLO) [16, 27]. Although both forms were shown to induce a potent E7-specific CTL response, only Lm-LLO-E7 induces regression in E7-expressing TC-1 tumor models. Hussain et al., have shown that the therapeutic differences in these two forms of Lm-based E7 vaccine was due to induction of functionally suppressive Tregs by Lm-E7 while Lm-LLO-E7 vaccine was shown to decrease the level of Tregs, with the CTL activity equally induced by both treatments [22]. Further mechanistic studies from our group with improved attenuated Lm-based E7 vaccine revealed that LmddA-LLO-E7 (Lm-LLO-E7) does not affect the absolute number of Tregs (CD4^+^FoxP3^+^); instead, the LLO component significantly enhances the number of CD4^+^FoxP3^−^ and CD8^+^ T cells, thus effectively decreasing the proportion of Tregs in the total number of T cells [17]. Our group also demonstrated that blocking immune inhibitory PD-1/PD-1 ligand signaling with anti-PD-1 Ab in the presence of Lm-LLO-E7 exhibits a synergistic effect with resultant therapeutic benefit in the TC-1 tumor model [15]. In that study, complete tumor regression was correlated with a Lm-LLO-mediated decrease in the percentage of Tregs within the CD4^+^ T cell pool and MDSCs, and an E7-mediated increase in the number of effector T cells, while blocking of PD-1 significantly enhanced expansion and tumor infiltration of antigen-specific CD8^+^ T cells. Moreover, it has been shown that depletion of both CD4^+^ and CD8^+^ T cells leads to abrogation of the anti-tumor activity of Lm-LLO-E7 [16].

The balance between immune effector CD4^+^ or CD8^+^ T cells and immune suppressive Tregs is critical for either protective or pathogenic immune response, hence determining the outcome of cancer treatment. Tipping the equilibrium between the effector T cells and Tregs towards the higher T effector:Treg ratio in the tumor microenvironment has been correlated with favorable outcome of tumor-specific immune response, hence a higher chance for the cure of cancer in both preclinical and clinical studies [9, 31–33]. The current study was executed with the hypothesis that *Listeria*-based immunotherapy will generate an antigen-specific immune response with a decreased population of suppressive cells, and stimulation with anti-GITR agonist mAb will further enhance the antigen-specific immune response and may subsequently lead to a profound anti-tumor effect.

To the best of our knowledge, we show for the first time that the combination of agonist anti-GITR Ab and *Listeria*-based immunotherapy leads to synergistic anti-tumor effect with prolonged survival of TC-1 tumor bearing mice. Using an adenoviral-E7-based vaccine, it has been shown that anti-GITR Ab enhanced the effects of the vaccine leading to complete and permanent eradication of tumors [34]. Significant increase in IFN-γ-producing T cells was observed when anti-GITR Ab was combined with the vaccine, however significant changes were not observed either in the density or the suppressive potential of Tregs in the peripheral blood leucocytes [34]. We demonstrate in the tumor microenvironment that *Listeria*-based immunotherapy when combined with agonist anti-GITR Ab enhances both the CD8^+^ T cells as well as the number of non-Treg CD4^+^ T cells resulting in enhanced therapeutic effector cell/suppressor cell ratios. Since, TC-1 is a Treg-depndent model [35] and it has been shown that the efficacy of GITR-targeting therapy requires depletion of Tregs [14], the increased therapeutic efficacy of the *Listeria*-based immunotherapy combined with anti-GITR Ab is dictated by the dilution of the Treg population. In addition, stabilization of the levels of suppressive MDSCs within the tumor microenvironment also play a significant role in the observed synergistic effects of this combination.

## Conclusions

Taken together, our studies highlight the importance of combining an immune response-inducing component with effector immune response enhancement and targeting suppressive mechanisms. We believe that our findings provide a promising and translatable strategy that can enhance the overall efficacy of cancer immunotherapy.

## Abbreviations

APC: Antigen presenting cell
CTL: cytotoxic T lymphocyte
CTLA-4: cytotoxic lymphocyte antigen-4
GITR: glucocorticoid-induced tumor necrosis factor receptor-related protein
GITR-L: GITR Ligand
LLO: Listeriolysin O
Lm: *Listeria monocytogenes*
MDSC: myeloid-derived suppressor cells
MHC: Major histocompatibility complex
PD-1: programmed death receptor-1
RBC: red blood cells
TNFR: tumor necrosis factor receptor
Tregs: regulatory T cells
